# Crystallized and fluid intelligence of university students with intellectual disability who are fully integrated versus those who studied in adapted enrichment courses

**DOI:** 10.1371/journal.pone.0193351

**Published:** 2018-04-23

**Authors:** Hefziba Lifshitz, Jay Verkuilen, Shlomit Shnitzer-Meirovich, Carmit Altman

**Affiliations:** 1 ID Majoring Program, Department of Special Education, School of Education, Bar-Ilan University, Ramat-Gan, Israel; 2 Educational Psychology and Public Health, The Graduate Center, The City University of New York, New York, New York, United States of America; 3 Levinsky College, Tel-Aviv, Israel; 4 School of Education, Bar-Ilan University, Ramat-Gan, Israel; TNO, NETHERLANDS

## Abstract

**Background:**

Inclusion of people with intellectual disability (ID) in higher postsecondary academic education is on the rise. However, there are no scientific criteria for determining the eligibility for full inclusion of students with ID in university courses. This study focuses on two models of academic inclusion for students with ID: (a) separate adapted enrichment model: students with ID study in separate enrichment courses adapted to their level; (b) full inclusion model: students with ID are included in undergraduate courses, receive academic credits and are expected to accumulate the amount of credits for a B.A.

**Aim:**

**(a)** To examine whether crystallized and fluid intelligence and cognitive tests can serve as screening tests for determining the appropriate placement of students with ID for the adapted enrichment model versus the full inclusion model. (b) To examine the attitudes towards the program of students with ID in the inclusion model.

**Method/Procedure:**

The sample included 31 adults with ID: students with ID who were fully included (*N* = 10) and students with ID who participated in the adapted enrichment model (*N* = 21). Crystallized and fluid intelligence were examined (WAIS-III, Wechsler, 1997) and Hebrew abstract verbal tests (Glanz, 1989). Semi-structured interviews were conducted in order to examine the attitudes of students in the inclusion model towards the program.

**Outcomes and results:**

The ANOVAs indicate that the most prominent difference between the groups was in vocabulary, knowledge and working memory. ROC analysis, a fundamental tool for diagnostic test evaluation, was used to determine the students’ eligibility for appropriate placement in the two models. Seven tests distinguished between the groups in terms of sensitivity and specificity. The interviews were analyzed according to three phases.

**Conclusions/Implications:**

The results indicate that students with ID are able to participate in undergraduate courses and achieve academic goals. The general IQ and idioms test seem to be best determiners for appropriate placement of students with ID to one of the two models. The included students with ID are motivated and self-determined in continuing in the program.

## Introduction

Despite the increasing trends of postsecondary education (PSE) for adults with intellectual disability (ID) in colleges and universities, studies to date have focused mainly on the social aspects of PSE [1], i.e. the attitudes of the traditional students and lecturers towards students with ID, the academic accommodations [2] and the impact of PSE on their employment [3], without relating to the cognitive aspect of inclusion. In all academic PSE programs, the students’ eligibility is based on educational and employment experiences, interests and motivation to undertake the course, and the support structures they have around them [2]. As Plotner and Marshall point out: "there are few if any research guidelines to help program developer, prepare and plan PSE" ([4],pp.59). There is no procedure of scientific criteria for determining the eligibility for full inclusion of students with ID in academic university courses [4]. Our study attempts to shed light on this issue.

### Intellectual disability

Traditionally, classification systems of the mental retardation (MR) revolved primarily around the range of IQ scores achieved by people who met the criteria of an IQ score two or more standard deviations (IQ 70–75) below the mean [5]. The most common classification scheme involved grouping people based on IQ into one of four subgroups: mild (IQ from 55–75), moderate (IQ from 40 to 55), severe (IQ from 25 to 40) and profound (IQ below 25). In 2010, according to the Rosa’s Law and the US federal law (Rosa’s Law) [6], the term "Intellectual disability" was confirmed as an alternative to "mental retardation". The traditional definition of mental retardation of the American Association of Mental Retardation [5] has been replaced by a new one defined by the American Association of Intellectual and Developmental Disability [7, 8] and by the Individuals with Disabilities Education Act [9, 10]. Intellectual disability is characterized by significant limitations in both intellectual functioning and in adaptive behavior including conceptual, social, and practical adaptive skills. This disability arises before age 18. The 2002 and 2010 American Association of Intellectual and Developmental Disability (AAIDD) classification manuals [7–8] suggested four levels of support (intermittent, limited, extensive, pervasive) as an alternative to the four IQ levels; however, these levels were not intended to form a classification system in the same way as the four levels of mental retardation (mild, moderate, severe, profound) and therefore the traditional classification system is still in use [8]. The definition of ID in the DSM-5 [11] is similar to that of the AAIDD from 2002. According to the DSM-5, the intellectual deficit of individuals with ID is largely manifested in a lack of understanding, reasoning, problem-solving, planning, abstract thinking, learning from experience and academic learning ([11], pp.33). Nevertheless, the classification system was revised again to include the four levels of ID (mild, moderate, sever and profound) which are based not on IQ, but on adaptive behavior skills.

### Postsecondary academic education for adults with ID

Despite the above-mentioned cognitive difficulties of individuals with ID, inclusion in postsecondary academic education (PSE) for adults with ID, with and without Down syndrome, is on the rise [4]. The universities of Alberta, Canada [1], were the first to open their doors to students with ID, followed by Flinders and Deakin universities in Australia [3]. In the US, there are 200 programs across 37 states for students with ID [4]. Many of these programs focus on social, vocational and life skills and students with ID study in separate courses tailored for their level [4]. Other programs have an academic orientation in which students with ID attend and audit selected undergraduate classes and receive a support system [1–2]. In all of these programs, students with ID receive a certification for auditing the courses.

As far as we know, there are three adults with Down syndrome (DS) in the world who succeeded in completing a bachelor’s degree: Aya Iwamo finished her BA in literature (Iwamo, Japan) [12], Pablo Pineda [13] finished his BA in education and works as an actor (Spain) and another student with Down syndrome completed a BA (USA) and works as an assistant to an English teacher in Germany (details are hard to track). The Empowerment Project at the School of Education at Bar-Ian University is a program that seeks to replicate these results and provide PSE for individuals with ID.

### Empowerment Project: Two models of inclusion of adults with ID at the Bar-Ilan University

UN Convention on the Rights of Persons with Disabilities states: "Parties shall ensure an inclusive education system at all levels and lifelong learning directed to: The full development of human potential, talent and creativity… sense of dignity and self-worth” [14]. In line with the UN agenda, The Machado Chair for Research and Human Development, School of Education at Bar-Ilan University launched the Empowerment Project, which is based on two models of university inclusion for adults with ID.

#### Model 1: Separate model—Adapted enrichment courses [4]

Students with mild ID attended the School of Education at Bar-Ilan University once a week for four academic courses adapted to their level, including: Developmental Psychology, Sociology, Geography, Self-advocacy, etc. The lecturers were MA students in the ID Program of the School of Education. The purpose of this stage is the acquisition of academic knowledge in various domains that may be relevant to their lives.

In the second year, the same students were included in a typical undergraduate research seminar on "Lifelong Learning of Individuals with Disability". As part of the seminar, students with typical development (TD) and students with ID study subjects related to self-advocacy. There was peer-learning between the two groups. The students with ID were exposed to the concept of research via the use of self-concept, hope and optimism questionnaires. Their task was to interview two friends with ID using the above questionnaires. They were taught to score questionnaires and even to insert the data into the Excel software, while the traditional students performed the statistical analysis of the data that the students with ID collected. Both groups analyzed the results, drew conclusions and presented their research, including theoretical background, method, results and discussion via a PowerPoint presentation.

The educational objectives were: to acquire knowledge on academic subjects that may be relevant to this population, to develop strategies for learning, to access the university’s libraries, to conduct small research projects and to use the computer lab. The social objectives were: to expose students with ID to the traditional students (with typical development) in class and during breaks, to expand the friendship circle of students with ID, to empower and strengthen their self-image, confidence, and quality of life, and to construct positive attitudes towards individuals with disability among the traditional students. The students received a certificate of participation upon completion of this project.

#### Model 2: Full inclusion with support [4]

After two years of running the adapted enriched model, we decided to administer a series of crystallized and fluid tests using the WAIS-III [15] and Hebrew abstract verbal tests [16]. As stated, there were no scientific criteria for determining the appropriate placement of students with ID for full inclusion in regular university courses, therefore, it was decided that students with ID who exhibited a general IQ of 60 and above would be assigned to the full inclusion model, whereas those who exhibited a general IQ under 60 would remain in the adapted enrichment model. Currently, 10 students with ID are included in undergraduate courses (four with Down syndrome, one with Williams syndrome, one with Kabuki syndrome and four with no specific etiology). To date, these students have completed the following undergraduate courses: "Introduction to Special Education", "Intellectual Disability", "Informal Education”, "Computers for Children with Special Needs", "Informal Education", "Theories of Special Education", "Strategies of Cognitive Modifiability" and "Judaism". These courses were chosen according to their relevance to the life of individuals with ID. The students were registered through the university as auditors (for these specific courses), which is a position that allows students to receive academic credits if they fulfill the requirements of the course. With the assistance of a special education teacher who accompanies the students during the courses (see Method section), the students participating in this project have so far received 11 academic credits (out of 64 credits needed for the BA degree) for completing the requirements for the courses. The ultimate goal of this project is to enable students with ID to complete the remaining 53 credits required for a B.A. Only students with ID who participated in Model 1 (enrichment courses), could move to the Model 2 (full inclusion); however, not all the students with ID who participated in Model 1 could move to Model 2 (see Method section).

### The theoretical basis for the Empowerment Project

The Empowerment Project was anchored in several theories: The Compensation Age Theory, the Structural Cognitive Modifiability (SCM) and the Cognitive Reserve theories.

*The Compensation Age Theory (CAT)* [17] postulated that chronological age (CA) plays an important role in determining the cognitive ability of individuals with ID, beyond their mental age (MA). The CAT claims that in later years there is compensation for the developmental delay experienced by individuals with ID in their early years. More specifically, their intelligence and cognitive performance may continue to increase until their 50s, thus modifying their ID at an advanced age. Furthermore, not only endogenous factors (age, etiology, IQ level), but also exogenous factors (lifestyle) influence cognitive functioning. Our vision for the Empowerment Project is to design a PSE experience that will enhance the cognitive functioning of adults with ID as postulated by the CAT and as was found by our previous research [18].

The Structural Cognitive Modifiability (SCM) Theory was at the base of the CAT. The SCM [19, 20] postulates that the human organism is a system open to its environment and accessible to change as a result of environmental intervention, even in the presence of three formidable obstacles usually believed to prevent change: age, etiology, and severity of limitation.

The Cognitive Reserve Theory (CRT) [21] states that living into old age in terms of cognitive functioning depends on the degree or quality of ‘reserve’ (remaining resources) in the brain. The CRT posits that higher cognitive reserve in the form of brain networks that are more efficient or have greater capacity in face of increased demands is what enables people to perform at higher levels of task difficulty. One might argue that individuals with ID have a lower cognitive reserve due to their lower level of intelligence, fewer opportunities for cognitive education and cognitive leisure activities. Our argument [17] was that the cognitive reserve of individuals with ID should be examined within the population with ID, and not compared to the general population.

Lifshitz and colleagues found that adults with ID can benefit from focused cognitive interventions aimed at strengthening specific cognitive skills that are prone to age deterioration, such as abstract verbal skills, orientation in time and space, memory [22], analogical reasoning [23] and metaphoric language [24]. In these studies, adults with ID gained more from mediation than adolescents with ID with the same cognitive level.

### Crystallized and fluid intelligence in adults with ID

McGrew [25] re-defined the Horn-Cattell model [26] of crystallized and fluid intelligence. Crystallized intelligence is defined as “a person’s acquired knowledge of the language, information and concepts of a specific culture” (25p.5). Crystallized intelligence is considered a “maintained” ability that increases into a person’s 60s and then declines. Fluid intelligence is defined as “the use of deliberate and controlled mental operations to solve novel problems that cannot be performed automatically” (25p.5). It is associated with frontal executive function [27], working memory, analogies and understanding of metaphors. Fluid intelligence is a “vulnerable” ability, peaking into one’s early 20s and then declining [25, 27]. The crystallized and fluid tests used in this study (see Method section) can be regarded as markers for these constructs.

Research on crystallized and fluid intelligence among adults with non-specific ID (NSID) and with Down syndrome is scant. In Devenny et al. [28], memory (which is considered a fluid test) of adults with NSID and Down syndrome improved until their 40s and was maintained until their 50s. Contrary to the crystallized and fluid evolution in the general population, Kittler, Krinsky-McHale, and Devenny [29] found deterioration in the verbal subtests of the WISC-R over a 7-year period among adults with ID. However, Facon [30] found a similar evolution of the WAIS-R [31] verbal and performance scales among adults with NSID and with Down syndrome and that of adults with typical development. In the present study, crystallized and fluid intelligence were examined by the WAIS-III [15] and by a series of Hebrew verbal-crystallized and fluid tests.

#### Goals of the study

This study examined crystallized and fluid intelligence and cognitive tests of the fully included students with ID (*N* = 10) compared to students with ID in the adapted enrichment courses (*N* = 21). The study’s goals were: (1) To delineate scientific criteria of eligibility for full inclusion of students with ID who participate in adapted enrichment courses, i.e. to examine whether a general IQ of 60 can be a good predictor of success of students with ID in undergraduate courses; (2) to identify the cutoff points in each of the intelligence and cognitive tests that is, to develop screening tests in order to better classify students who are most appropriate for the full inclusion model, and whether certain students should not move beyond the adapted model; (3) to examine the attitudes towards the program of students with ID who are fully included in undergraduate courses.

## Materials & methods

### Participants

The sample included 31 adults with ID who participated in the postsecondary Empowerment Project, which is a two-model academic inclusion project for adults with ID offered by the School of Education, Bar-Ilan Ufniversity (BIU). The students were recruited to the program by the Israel Association of Persons with Down Syndrome and by the Division of ID in the Israeli Ministry of Social Affairs and Social Services. Due to a lack of scientific criteria for eligibility of adults with ID to participate in PSE [2], the decision on their adaptation in PSE was based on their level of ID according to the DSM-5 [11], their reading ability and their interest in participating in PSE. Participants met the following criteria: Adults with mild ID [11], who knew to read and write, were independent in Activities of Daily Living (including arriving at the university campus by public transportation independently), without maladaptive behavior. We did not administer an intelligence test at the beginning of the program in order to preclude and biases. All of the participants worked in vocational centers or in the open market in the morning (with no significant differences between the fully included and those who study in adapted enrichment courses) three times a week and participated in leisure activities in the afternoons. As previously stated, all of the participants could read, but their level of reading or comprehension was not assessed. The current sample was divided into two groups according to the above-mentioned models:

Etiology: Of the ten students in the full inclusion model, four were persons with Down syndrome, one women has Williams syndrome, one woman has Kabuki syndrome and the other four are persons with ID with a non-specific etiology (NSID). Of the students studying in the adapted enrichment model, eight have Down syndrome and 13 have NSID.
Separate adapted enrichment model: 21 (68%) Students with ID participated in adapted enrichment courses (henceforth: the adapted enrichment group, Chronological age (CA) = 26–40; Mean CA = 35.79, *SD* = 6.86). They attend the university once a week for four academic hours. The courses were adapted to the level of the participants by the traditional MA students of the School of Education at the Bar Ilan University. In addition to the academic knowledge imparted in the courses, we worked on cognitive skills, such as working and long-term memory, executive function and meta-cognitive skills, self-regulation skills such as learning for an examination, preparing homework and self-management skills, i.e. searching in the web and using technological devices such as laptops, etc. In the separate adapted enrichment model, students with ID received a certificate of participation in this project. All participants in this group reside in residential facilities of adults with ID under the supervision of the Division of Intellectual Disability of the Israeli Ministry of Social Affairs and Social Services. The students in this group studied in special education schools.
Full inclusion model: 10 students with ID (32%) were fully included in undergraduate courses (CA = 26–51; MCA = 31.14, *SD* = 5.84, with no significant difference in CA between those in the adapted model versus those in the inclusion model, *t*(28) = 1.64, *p* > .05). These participants lived at home with their parents. Three students studied in special education schools and four in regular schools.

The process of identifying of the students with ID who were assigned to the full inclusion model: After two years of running the adapted enrichment program, we identified 13 relatively highly capable students. Their higher level was expressed mainly in their advanced understanding, verbal expression and memory in comparison to the other 21 that participated in the adapted enriched program. At this point, we decided to administer the intelligence and cognitive battery. The purposes were (a) to establish criteria that will enable us to determine the eligibility for participation of students with ID in the full inclusion versus the adapted enrichment models and (b) to identify future candidates who may be suitable for either program. One might argue that since we administered the tests two years after starting the adapted courses program, their previous education may have impacted the results; however, our claim is that the students with ID in the adapted enrichment model also learned academic material, which could have similarly influenced their IQ level as well as IQ of the students in the inclusion model.

The findings indicated that the 13 highly capable students exhibited general IQ of 60 and above, while the remaining 21 participants in the adapted enriched program exhibited an IQ of 50–60. It was decided that students with ID who exhibited a general IQ of 60 and above would be assigned to the full inclusion model, whereas those who exhibited a general IQ under 60 would remained in the adapted enrichment model. According to this criteria, 13 students with ID were assigned to the full inclusion model; however, three of them were excluded after the preparatory program due to behavioral-emotional problems and inability to complete course requirements. At this point, we found out that intelligence is not the only criteria of eligibility for the participation of persons with ID in full inclusion, but emotional maturity over time should also be taken into consideration. We excluded the three students with ID from the study.

The criteria for participation in the full inclusion model: Participation for two years in the adapted enrichment model, an IQ of 60 and above, and emotional maturity with no mal adaptive behavior.

Preparatory program towards full inclusion and learning strategies: Prior to the academic year, the students with ID who were assigned to the full inclusion model participated in a two month preparatory program which aimed to prepare them for their inclusion in undergraduate courses. To achieve this goal we used the Universal Design for Learning strategies (UDL, CAST) [32] with adaptations to students with ID. The UDL has defined and adopted three main principles for learning: (a) Provide multiple means of representation: The aim is promote resourceful, knowledgeable students by providing options for comprehension, language and perception. We combine this principal with Bloom’s taxonomy for population with ID [33] for population with ID [33] (b) Providing multiple means of actions and expression: The aim is to promote strategic, goal directed students by providing options to experience executive function, options for expression and communication and providing options for experience self regulations. Easy to read—Making written information easier to understand for people with learning disabilities [34], (c) Providing multiple means of engagement: The aim is to promote purposeful, motivated students by providing options for Self regulation, sustaining efforts and persistence and for recruiting interests. The academic skills, strategies and cognitive process are presented in Appendix 1.

Support system during the courses: The 10 participants in the included group were divided into two groups of five students. Each group studied in different courses and had a special education teacher accompanying them. We trained two special teachers to work with the students with ID according to Universal design learning [32]. For each academic hour in the university course they received an additional academic hour of preparation from the special education teacher. Prior to the beginning of the course, the special education teacher got the curriculum of the courses and the content of every lesson from the lecturers. A reader was composed for each course, which included relevant reading materials for the course. In that way the teachers could prepare the students with ID towards the next lesson in every course. During these preparatory courses, the teachers worked with the students with ID according to the didactic strategies and cognitive process mentioned above. They also prepared the students with ID towards exams and helped them in performing the course work.

The criteria for choosing the undergraduate courses: The pedagogical committee of the project chose courses from the social sciences which are based on verbal knowledge and that has content that is relevant to the world of adults with ID, such as "Introduction to Special Education" (2 credits), "Intellectual Disability" (1 credit), "Computer Programming for Special Needs" (1 credit), "Informal Education" (2 credits), "Theories of Special Education" (2 credits), "Strategies of Cognitive Modifiability" (1 credits) and "Judaism" (2 credits). All courses were applicable and relevant for this population by dealing with special education and informal education, the rationale and values of leisure activities and the residence framework for individuals with disabilities, Jewish humanistic values and their implication to everyday life, and constructing educational programs for special populations. This fact served as a scaffolding for their self-efficacy to cope with these courses. They participated in the course actively and shared their life experience as persons with disability. The lecturers at this courses received training in using the ULD and the other strategies mentioned above.

Criteria of success of the students with ID in the regular undergraduate courses: In some courses, the final score is determined by exams and in some, the final score is determined by course task. The criteria required of the students with ID in the traditional undergraduate courses were the same criteria that were required from the traditional students. In Israel, the scale scores in exams range between 0–100 with a passing score of 60.

In some courses the exam is a multiple choice questionnaire which was difficult for students with ID. We developed a method of teaching them to answer multiple choice questions. Their results which followed our teaching improved as captured by the students’ scores which ranged between 60–70. In courses in which there were no exams, the final score is comprised of course assignments: group work during class, reading articles and presenting them, course work and the combination of these tasks. Course works’ score is comprised of strict criteria of expressive written ability, using scientific terms, and reading academic articles. The same criteria are used for examining the students with ID. to these criteria the scores of the students with ID range between 65–80. Up to date, they earned 11 academic credits.

### Assessment materials

Crystallized and fluid intelligence were examined by the Wechsler Intelligence Test for Adults, WAIS-III^HEB^ [35] that represents crystallized and fluid intelligence skills in a typical population. In addition to the IQ scores and the verbal/perceptual scales, four other indexes were derived from the verbal and performance sub-scales: Comprehension index composed of vocabulary, similarity and knowledge, Perceptual index composed of picture completion, block design and matrices, Memory index composed of arithmetic, digit forward and backward, series letters and numbers and Speed processing index composed of coding and signs.

The crystallized and fluid tests battery were examined using the MANN—Abstract Verbal Thinking Test [16] which examines verbal abstract thinking and verbal intelligence abilities. Four tests were found to be appropriate for the population with ID [36, 37]: synonyms, classification, contrast and verbal analogies. Semantic and phonemic fluency tests [38] were also administered, as well as Homophone Meaning Generation Test*–HMGT* [39].

The results of a confirmatory factor analysis with Varimax rotation confirmed two different clusters (the minimum loading set point was .35). The first type contained the following tests: semantic fluency, synonyms, contrast, and classification which accounted for 41.39% of the variance. The second type contained the following tests: phonemic fluency, verbal analogy, Homophone Meaning Generation Test—HMGT and idioms tests which accounted for 37.59% of the variance. Three academic scholars (one is an expert in the Hebrew language, and two are experts in psychology) confirmed the division into two clusters with an inter-rater reliability of 90%. The tests in the first cluster are of the crystallized type, and their solution involved cultural and environmental experience and knowledge based on what can be acquired through life experience. The second cluster was of the fluid type. Although these tests were also verbal, they demanded the use of deliberate and controlled mental abstract operations which could not be performed automatically and could not be acquired through the environment [25]. Table 1 presents the rotated component matrix of the different tests.

**Table 1:** Rotated component matrix of confirmatory factor analysis on the different Hebrew verbal tests.

	Factor 1	Factor 2
Contrast	.90	
Synonyms	.88	
Classify	.85	
Semantic fluency	.63	
Analogy		.88
Phonemic Fluency		.87
HMGT		.86
Idioms		.62

The crystallized battery: The Synonyms test (12 items) [16] examines verbal abstract thinking or verbal intelligence abilities, and is highly correlated with the Verbal Wechsler intelligence tests. The participants were presented with a key word and were asked to find a similar word from a list of five other words (e.g., wall: gate, path, way, balcony, side). Correct answers were given 1 point (range 0–12; test-retest reliability = .90; α = .91). Classification (12 items) [16] is aimed at assessing comparative behavior. The participants are presented with a list of four words and have to find a concept or a single trait that best characterizes the list (e.g., love, hate, worry, joy: trips, voices, liquid, feelings). A correct answer was given one point **(**α = .72). Contrasts (12 items) [16]: The participants have to find the opposite word out of five alternatives (e.g., floor: ceiling, bag, cabinet, wall, balcony). A correct answer (range 0–12) was given one point (test-retest reliability = .82). Semantic fluency [38]: The number of words generated in one minute for each of the following three semantic categories: animals, fruits and vegetables, was obtained. The score is the sum of the words generated for all three categories (test-retest reliability = .86).

The fluid battery: Phonemic fluency test [38]: This test requires the ability to deal with novelty and is a culturally unbiased nonverbal test [27]. The number of words generated in one minute for the letters bet (/b/), gimel (/g/), and shin (/š /) was obtained. Instructions were as follows: “I want you to say as many Hebrew words as possible that begin with a certain letter”. The score is the sum of the words generated for all three letters (test-retest reliability = .86; α = .91). Verbal analogies (12 items) [16] examines the understanding of verbal analogies. The participants are presented with a pair of words (key pair—A:B) and a list of five words that can relate to a C term in a second pair in the same way that the words relate in the key pair (e.g., hat is to head as shoe is to: arm, eyes, ears, foot, and forehead). A correct answer was given one point (rang 0–12) (test-retest reliability = .80). Homophone Meaning Generation Test—HMGT [39] examines the ability to shift between the different meanings of a homophone (10 items in Hebrew). The participants were instructed to say all meanings of the homophone, e.g. "He wrote a letter". A correct answer was awarded one point (range 1–10) (test-retest reliability = .83). Idiom Comprehension (12 items) [39] examines understanding of figurative meanings of idioms (20 items, e.g., "he got cold feet"). Four interpretations were offered: a correct idiomatic interpretation; a literal interpretation; a literal distracter; an unrelated interpretation. Correct answers were awarded one point (range 0–20) (test-retest reliability = .71).

### Qualitative analysis—Semi-structured interviews

A qualitative method of semi-structured interviews [40, 41] was carried out among students with ID in the full inclusion model (three years after participating in the full inclusion model). The interviews enabled in-depth understanding of the meanings, perceptions and feelings of the students with ID towards studying in the university in regards to coping with successes and failures. They were asked the following four questions; “Is studying in undergraduate courses at the university important to you? Why?", "How do you cope with difficult tasks?", "What is the contribution of the full inclusion program to your life?", "Would you like to continue in the program and why"?

Content analysis [42, 43] was performed according to three stages of qualitative research according to Shkedi [43] as follows: (a) Deriving simple categories as well as sub-categories according to participants’ answers, (b) Mapping the categories (understanding the relation and the association between categories and deriving new categories), and (c) Generating the core category or constructing theoretical themes. Three judges analyzed the answers: one academic scholar in the field of ID, one academic scholar in the field of qualitative research and the head of the ATID—Israeli Association for Individuals with Down Syndrome. A 90% inter-rater reliability was found.

### Procedure

Authorizations were obtained from the Ethics Committee School of Education Bar Ilan University and the Division of Individuals with ID in the Ministry of Social Affairs and Social Services who approved the participants’ consent. In addition, written consent for participation was obtained from the participants’ parents/guardians. All participants in the adapted model and inclusions groups signed an adapted informed written consent form for participation in program (the adapted or full inclusion models. We clarified that they can quit the program whenever they want. The study’s aim and procedure were explained to all participants, who signed an adapted informed written consent form for participation in scientific research. We took the original consent forms in studies where typically developing students took part in and adapted it with the rules of Easy to Read [34] for participants with ID. Participants read and signed the consent form. We also orally clarified that there is no obligation to participate in the study. According to the Normalization principal [44] for students who participate in scientific studies, the participants in this study chose payment or a gift for investing their time and effort.

The WAIS-III was administered in a quiet room by a psychologist who is an expert in the field of ID at the School of Education, individually for each participant for about 90 minutes. The aforementioned Hebrew MANN verbal intelligence tests [16], as well as the homophones, semantic and phonemic fluency were administered individually to each participant by MA students in ID in a small room at the School of Education. This stage was conducted in two sessions which lasted about 45 minutes, with a 15-minute break between sessions. After three years of participating in the full inclusion model, we conducted the semi-structured interview with each participant which lasted one hour. The interviews were held by MA students in ID who has expertise in conducting interviews.

#### Statistical analysis

Crystallized and fluid data were analyzed using parametric statistical procedures, e.g. ANOVAs, and non-parametric procedures such as the ROC analysis. A one-way MANOVA’s with the two groups (integration/enrichment) as the independent variables and the different subscales as dependent variables was carried out.

The ROC (Receiver Operating Characteristic) curve was used in order to determine the diagnostic metrics of crystallized and fluid variables evaluation [45]. In our case, for instance, the IQ intelligence test was evaluated in an attempt to decide the adaptation of a student with ID to the full inclusion versus the adapted enrichment model of inclusion in the academic world (see Zweig and Campbell [45] for technical results relevant to the ROC analysis).

In a ROC curve, the true positive rate (sensitivity) is plotted as a function of the false positive rate (1-specificity) for different cutoff points of a parameter. Each point on the ROC curve represents a sensitivity/specificity pair corresponding to a particular decision threshold. The area under the ROC curve (AUC) is a measure of how well a parameter can distinguish between two diagnostic groups (i.e., students with ID participating in the full inclusion versus those who study in the adapted enrichment program). AUC has a value between 0 and 1, with higher values indicating greater distinction between groups. An AUC = 0.5 indicates chance performance, essentially no better than randomly allocating participants to groups while an AUC = 1 would indicate perfect distinction. In this study, we consider measures with AUC greater than 0.8. We also examined the bootstrap confidence intervals for it, which are the most accurate for small samples, ensuring that the measure at least does not drop below 0.5.

For a particular cutoff, *sensitivity* measures the probability of the test, indicating that a student is suitable for the full inclusion program (given that they fulfilled all course requirements—tasks and final examination), while those in the adapted enrichment program are suitable for their program according to the tasks that are given in this course. *Specificity* measures the probability of the test indicating that a student would be unsuitable for the full inclusion program given that they did not fulfill their course requirements (e.g., failed the course examination and/or course tasks), while those in the adapted enrichment program are not suitable for the program because they exhibit higher scores in the screening tests and could be moved to the full inclusion group. A good test is both sensitive and specific. We also consider the percent of correct classification, which is an overall measure of performance for a given cutoff. A good cutoff generates a high percent of correct classification.

For every possible cutoff point or criterion value selected for discriminating between two populations, there are some cases with which the cutoff point of each test for the inclusion model will be correctly classified as true positive (.i.e., the student is suitable for the program), or classified as false negative (i.e., the student is not suitable for the program). On the other hand, some students who are in the adapted enrichment model will be correctly classified as true negative (i.e., the student is not suitable for the program and can move to the full inclusion program), but some cases in this group will be classified as false positive (i.e., the student is suitable for the adapted enrichment program).

## Results

Due to the small sample size, Shapiro-Wilk analyses were performed for each of the dependent variables. The findings indicated that all dependent variables were normally distributed in both groups (all *p*s > .05). Parametric analyses were therefore performed for each group separately.

### Differences in WAIS-III intelligence test and the cognitive battery among students with ID in the full inclusion and the adapted enrichment groups

**Fig 1:**
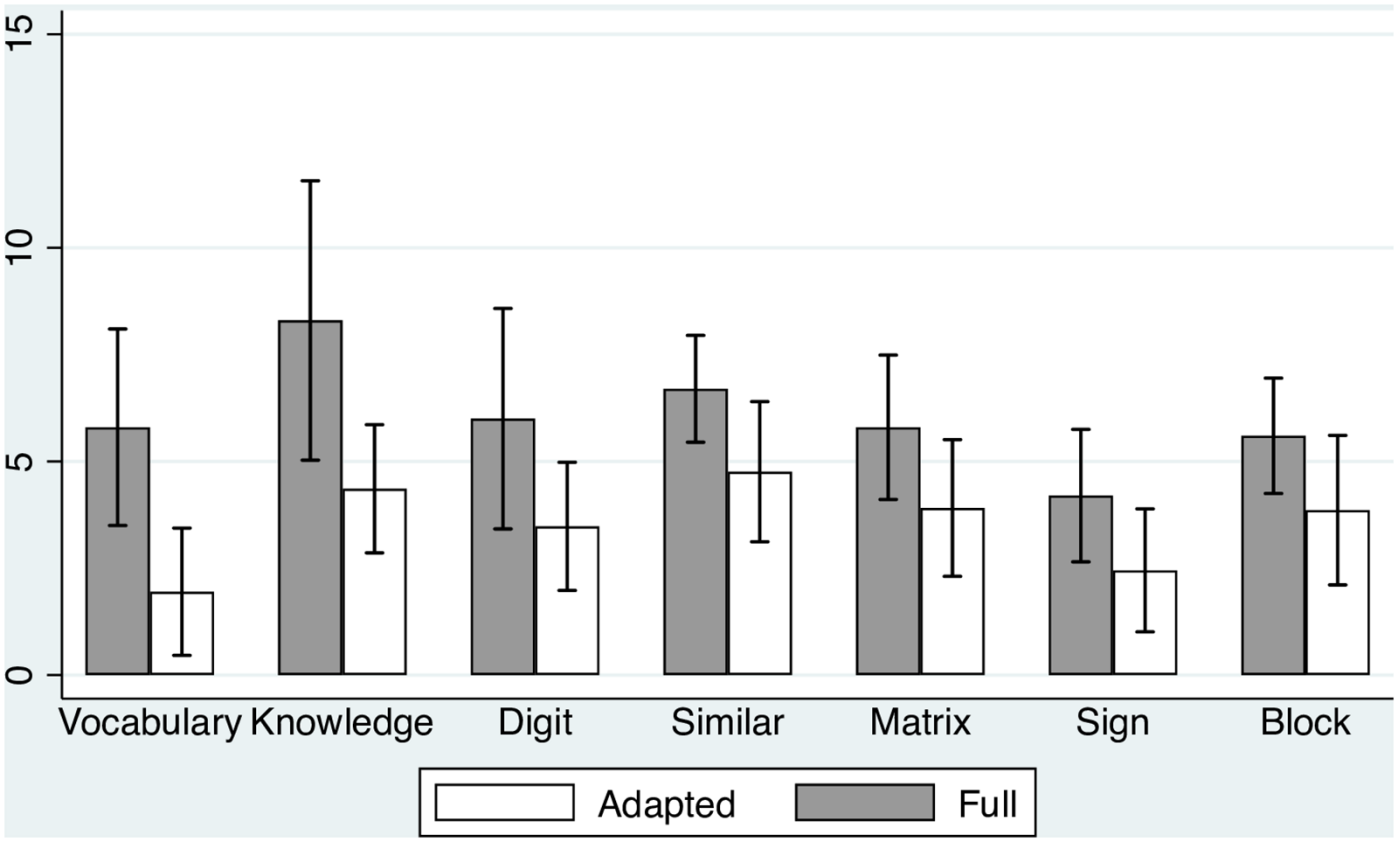
Hierarchy of the verbal and perceptual IQ subscales in the full integration and adapted enrichment groups.

We were eager to elucidate the differences between the students in the full inclusion model and those in the adapted enrichment program, beyond general IQ scores. In order to determine the differences between the full inclusion versus the adapted enrichment groups in the WAIS-III intelligence tests, a one-way MANOVA was performed with the two groups (inclusion/enrichment) as the independent variable and performances in the five verbal and five perceptual subscales as dependent variables. Significant differences in the WAIS subscales were found, *F*(10,19) = 5.76, *p* < .001, η_p_^2^ = .75, indicating higher performances in the full inclusion group compared to the adapted enrichment group. Means, *SDs* and *F* values of the full scale of the WAIS five verbal and performance IQ subscales are presented in Table 2 and Fig 1. A one-way MANOVA was also performed, with the two groups (inclusion/enrichment) as the independent variable and performances in the four IQ indexes as dependent variables. Significant differences in the IQ indexes were found, *F*(4,24) = 11.88, *p* < .001, η_p_^2^ = .66, indicating higher performances in the full inclusion group compared to the adapted enrichment group.

**Table 2:** Mean, SD, and *F* values of the WAIS IQ tests in the full inclusion (*N* = 10) and adapted enrichment (*N* = 21) groups.

	Full Integration		Adapted enrichment				
Test	*M*	*SD*	*M*	*SD*	*F*	*p*	*η2*
General IQ	68.70	4.24	56.59	3.94	62.05***	<.001	.67
Verbal IQ	72.90	6.59	59.68	4.69	42.21***	<.001	.58
Performance IQ	68.00	5.46	60.64	5.47	12.47***	<.001	.29
***Verbal subscales***							
Vocabulary	5.80	2.30	1.95	1.49	32.26***	<.001	.52
Similar	6.70	1.25	4.76	1.64	10.87**	.01	.27
Knowledge	8.30	3.27	4.36	1.50	22.32***	<.001	.43
Arithmetic	2.90	1.45	1.91	1.34	3.57	.07	.11
Digit span	6.00	2.58	3.48	1.50	11.89**	.01	.29
***Perceptual subscale***							
Picture completion	3.80	1.23	3.00	1.41	2.37	.13	.07
Block design	5.60	1.35	3.86	1.75	7.68**	.01	.20
Matrix	5.80	1.69	3.91	1.60	9.29**	.01	.24
Coding	4.00	0.82	3.36	1.29	2.03	.16	.06
Sign	4.20	1.55	2.45	1.44	9.66**	.01	.24
**Indexes**							
Comprehension index	82.33	10.69	62.55	10.31	22.35***	<.001	.45
Perception index	70.67	5.85	66.80	9.19	1.33	.26	.05
Memory index	69.22	12.76	53.60	4.22	24.90***	<.001	.48
Speed index	70.33	3.77	64.85	5.18	8.07**	.01	.23

*p < .05,

**p < .01,

***p < .001

The findings indicated that students with ID in the full inclusion group exhibited significantly higher scores in general IQ (IQ = 68.70; *SD* = 4.24;IQ = 56.59; *SD* = 3.94) for the full inclusion and adapted enrichment groups, respectively), in the verbal IQ (IQ = 72.90; *SD* = 6.59; IQ = 59.68; *SD* = 4.69 for the full inclusion and adapted enrichment groups, respectively) and in the performance IQ (M = 68.00, *SD* = 5.46;M = 60.64, *SD* = 5.47 for the full inclusion and adapted enrichment groups, respectively).

The Verbal IQ subscales: A one-way ANOVA was performed for each verbal IQ subscales. The most prominent difference (according to the effect size and SD) between the groups lies in vocabulary, followed by knowledge, digit span backward and forward and similarities. Thus, vocabulary, knowledge and working memory are the most prominent subscales that differentiate between the groups.
The performance IQ subscales: A one-way ANOVA was performed for each verbal IQ subscales. The most prominent difference between the groups according to the effect size and (SD) lies in Signs, Matrix and Block design. Thus, the most prominent differences between the full inclusion and the adapted enrichment groups lie in the verbal ability, followed by working memory. In addition, significant differences were found in Signs—a measure of speed as well as in Matrix and Block design.
Differences in the indexes: A one-way ANOVA was performed for each index, indicating that the most prominent differences between the students in the full inclusion group lies in the comprehension index (*M* = 82.33, *SD* = 10.69; *M* = 62.55, *SD* = 10.31 for the full inclusion and adapted enrichment groups, respectively), memory index (*M* = 69.72 *SD* = 12.26; *M* = 53.60, *SD* = 4.22 for the full inclusion and adapted enrichment groups, respectively) and the speed index. However, no significant differences were found between the two groups in the perceptual index.
The crystallized and fluid tests: A one-way MANOVA was performed with the two groups (inclusion/enrichment) as the independent variable and the four crystallized tests as dependent variables. Significant differences in the performances on the crystallized tests were found, *F*(4,24) = 5.96, *p* < .01, η_p_^2^ = .51, indicating better performance in the full inclusion group compared to the adapted enrichment group.

A one-way MANOVA was performed with the two groups (inclusion/enrichment) as the independent variable and performances in the four fluid tests as dependent variables. Significant differences in the performances on the fluid tests were found, *F*(4,24) = 3.97, *p* < .05, η_p_^2^ = .52, indicating better performance in the full inclusion group compared to the adapted enrichment group. Means, SDs and F values of the crystallized and fluid tests are presented in Table 3.

**Table 3:** Mean, SD, and *F* values of the crystallized and fluid tests in the full inclusion (*N* = 10) and adapted enrichment (*N* = 21) groups.

Crystallized tests							
Full Inclusion			Adapted Enrichment				
	*M*	*SD*	*M*	*SD*	*F*	*p*	*η*^*2*^
Semantic fluency	42.00	5.94	29.42	9.50	14.41***	<.001	.35
Synonyms	8.50	2.68	4.42	2.27	18.73***	<.001	.41
Contrast	7.40	2.27	4.32	2.38	11.33**	.01	.30
Classification	10.60	0.97	8.56	1.89	10.15**	.01	.28
**Fluid tests**							
Analogy	7.20	2.62	4.16	2.34	10.22**	.01	.27
HMGT	14.90	3.90	12.17	3.78	3.29	.08	.11
Idioms	15.44	3.05	7.75	4.43	19.92***	<.001	.51

*p < .05,

**p < .01,

***p < .001

Separate analyses for each of the crystallized and fluid subscales indicated significant differences in all four crystallized tests: semantic fluency, synonym, contrast and classification (Table 3). Significant differences were also found in three of the fluid tests: phonemic fluency, analogies and idioms.

### The cutoff points of intelligence and cognitive tests of the full inclusion and the adapted enrichment groups

ROC analysis was performed in order to consider the cutoff point in each of the intelligence tests and the Hebrew crystallized and fluid tests. We considered three measures: the sensitivity (probability that a student with ID in the full inclusion group would be correctly identified), specificity (probability that a student with ID in the adapted enrichment group would be correctly screened), and the Area Under the Curve (AUC) as an overall measure.

Sensitivity and specificity are conditional probabilities that show the value of a measure for identifying positive and negative cases, respectively. We consider 0.80 as a good value for AUC, sensitivity and/or specificity. Of the 24 tests, seven exhibit highest sensitivity and specificity (sensitivity and/or specificity above .8), as seen in Table 4.

**Table 4:** The AUC, sensitivity and specificity values for each variable.

Variable	AUC	Sensitivity	Specificity
**General IQ (≥ 66)**	.968	90%	100%
**Verbal IQ (≥ 66)**	.945	90%	86.36%
**Comprehension Index (≥ 72)**	.933	80%	81.82%
**Vocabulary (≥ 3)**	.9	90%	77.27%
**Knowledge (≥ 6)**	.822	80%	81.82%
**Synonyms (≥ 6)**	.836	80%	73.68%
**Idioms (≥ 14)**	.907	88.89%	91.67%

AUC = Area under curve

The seven variables that had AUC, sensitivity and specificity that distinguished between the two distributions overall were: IQG, IQV, comprehension index, vocabulary, knowledge, synonyms and idioms (Table 4). Table 5 presents the number of persons in the full inclusion model and in the adapted enrichment model who fell within the cutoff points as indicated by the ROC curve analysis in these tests.

**Table 5:** Percentage above and below the ROC cutoff point in the full integration (*N* = 10) versus the adapted enrichment (*N* = 21) groups.

	Inclusion positive	Inclusion Negative	Adapted Enrichment positive	Adapted Enrichment Negative
	Above cut off % N	Under cut off % N	Under cut off % N	Above cut off % N
**General IQ**	90%(N = 9)	10% (N = 1)	100% (N = 21)	0% (N = 0)
**Verbal IQV**	90% (N = 9)	10% (N = 1)	85% (N = 18)	14% (N = 3)
**Comprehension Index**	90% (N = 9)	10% (N = 1)	85% (N = 18)	14% (N = 3)
**Vocabulary**	90% (N = 9)	10% (N = 1)	81% (N = 17)	19% (N = 4)
**Knowledge**	90% (N = 9)	10% (N = 2)	85% (N = 18)	14% (N = 3)
**Synonyms**	80% (N = 9)	20% (N = 2)	85% (N = 18)	14% (N = 3)
**Idioms**	90%(N = 9)	10% (N = 1)	95% (N = 20)	4.8%(N = 1)

Nine of the ten students with ID in the full inclusion group matched the profile suggested by the ROC curve analysis (Table 5). The tenth participant in the full inclusion group, an individual with Down syndrome, did not seem to fit well from the beginning, and had difficulties throughout the course. In synonyms, two participants (both with Down syndrome) scored below the cutoff point.

Greater variation was found in the adapted enrichment group. None of the participants in the adapted enrichment group scored above the cutoff point in general IQ, making them distinctly different from the full inclusion group. Idioms produced only one participant above the cutoff point. As for verbal IQ, Comprehension Index, Knowledge, three participants scored below the cutoff point; however, these were not the same participants in the various tests. This variation in the number of the participants who scored above the cutoff in the various tests may be due to individual differences between participants with ID and shall be explored further in the discussion. The vocabulary index produced four participants above the cutoff point. It should be noted that in all cases, there was close to 90% precision in the full inclusion group and above 80% precision among the adapted enrichment group. Thus, the most reliable variables are the general IQ and idioms.

In conclusion, the findings revealed two ways of differentiating between students in the full inclusion group and those in the adapted enrichment group. The MANOVAs revealed significant differences between the groups in almost all subscales of the intelligence tests and the crystallized and fluid tests. It also ranked the differences between the groups. ROC analysis determined the appropriate placement of the students in the full inclusion group and the adapted enrichment group as well as those who score below the cutoff point in the full inclusion group and those who scored above the cutoff in the adapted enrichment group. IQG, IQV, comprehension index, vocabulary, knowledge, synonyms and idioms had high specificity and sensitivity which may further our understanding of the cognitive and language resources that enable full inclusion of students with ID.

### Analysis of the qualitative interview

Semi-structured interviews yielded several themes- their thoughts about the program, feelings of failure and success, and their way of coping with obstacles- related to the attitudes of participants to the program. The mapping stage according to Shkedi [43] revealed that the perception of undergraduate courses that our participants took can be explained by the three components of attitudes [46, 47]: cognitive, emotional and motivational (a combination of cognition and behavior, see Discussion section) components. Table 6 presents samples of the answers.

**Table 6:** Sample answers on the semi-structured interview.

	Cognitive answer	Emotional answer	Motivation (cognitive/ behavioral) answer
Is the studying in academic courses at the university, important to you and why?	- Personal growth -Expansion of knowledge - Contribution to my occupation -Desire to learn	-I am happy. -I am enjoying myself. -I feel good. -I like to learn.	- It broadened my knowledge - I learnt new things - I grew - I progressed at work
How do you cope with difficult tasks?		-When it is too difficult, I become nervous. -At the beginning it was hard, now I feel better. -I feel frustrated but I keep working. - I am enjoying myself. -Challenging tasks give me a good feeling.	-I tried to solve the problem alone (cope by myself) and if I don’t succeed, I ask for help from the teacher or my parents. -I prefer to seek for help. -I like challenges.
What is the contribution of the full inclusion program to your life?	-Improve my status in society - Improve my status at work -Enrich my knowledge	-I feel more strength. - I improved my self- image. -I improved my self- confidence. -I feel more respectable and dignified.	
Do you want to continue in the program and why?	Most people at my age study at the university.	-I love to learn. -I feel happiness. -I am enjoying myself.	-I want to broaden my knowledge. -I want to keep moving forward. -I want a BA degree. -As long as I can, I want to keep developing and progressing.

## Discussion

Three issues are at the core of the discussion: (a) Attribution of the different patterns in intelligence and crystallized and fluid tests between the groups (according to the MANOVA’s), (b) The criteria for determining the eligibility of the students with ID for the two models of inclusion in the academic world: the adapted enrichment model and the full inclusion model (according to the ROC analysis), (c) The cognitive, emotional and motivational attitudes of the included students with ID to their program.

### The difference in intelligence and cognitive battery between the adapted enrichment and the full inclusion groups

The findings indicate that the most prominent difference between the students who are fully included and those who study in the adapted enrichment model is the general IQ, which exceeds the 68 mark in the included group, compared to 56 mark in the adapted enrichment group. Significant differences were found in the verbal IQ of the full inclusion group which crossed the 72 mark (the range of three participants was 77–81), while the verbal IQ of the adapted enrichment group was approximately 59. The perceptual IQ of the full inclusion group was 68, compared to 60 in the adapted enrichment group.

In our opinion, the differences in verbal and perceptual subscales between the groups represent the developmental approach as well as the statistical approach and beyond the goals of this study, are important in understanding the nature of ID. The *developmental approach* is ecological, dynamically oriented and represents the progress of individuals with ID primarily in domains that that can be developed by their engagement with environmental challenges. Crystallized intelligence (verbal IQ) is considered to be culturally-dependent [25, 27, 48]. Vocabulary and knowledge are acquired through educational and leisure opportunities as well as via life experience. The *statistical approach* relates to the deviation from the statistical mean of the population. It reflects the fluid intelligence, which is more associated with general intelligence *g* [49] and expressed by the ability to solve novel problems and situations. Thus, the differences between the groups in both types of intelligence represent a developmental as well as a statistical gap.

Our participants were defined as individuals with mild ID according to new AAIDD ID definitions [7, 8] and the DSM -5 [11]. All students in the full inclusion group exhibited higher verbal IQ, and in the Hebrew crystallized tests [16], i.e. synonyms, semantic fluency, contrast and classification (One student with William syndrome (characterized by higher verbal ability) [50] and a student with NSID received 81 in the verbal IQ, while another student with Kabuki syndrome received 77). The difference in the crystallized tests between the groups could be attributed to the type of residence and learning environment.

All students in the full inclusion model lived at home with their parents while most of the students with ID in the adapted enrichment model lived in community residences. Home culture is more promising and provides more opportunities for nurturing the cognitive ability than community residence. Furthermore, three students in the full inclusion model studied in special education schools and seven in regular schools, while all students with ID in the adapted enrichment model studied in special education schools. Thus, the environment culture (type of residence and school) could be the causal factor in the difference in verbal abilities between the included students with ID versus those who study in the adapted enrichment model.

Scientists asked whether academic knowledge is domain specific, i.e. correlated with crystallized and/or fluid intelligence tests. Ackerman [51] examined the contributions of fluid and crystallized intelligence in predicting individual differences in academic knowledge between middle-age adults (age 21–62) who earned a B.A in exact sciences versus social sciences. It was found that crystallized intelligence had a considerable explanatory power in predicting knowledge in the social sciences, whereas fluid intelligence predicted knowledge in exact sciences. The gap in verbal scores found in our study among students with ID who are fully included, versus their peers who study in the adapted enrichment courses, supports the same effect that was found in populations with typical development [51]. The students with ID in the current study are fully included in education courses at the School of Education (Bar-Ilan University) which falls under the umbrella of the Faculty of Social Sciences and Jewish courses, under the broader category of humanism. Thus, their higher crystallized intelligence ability represent a normal phenomenon for typical students who study in social science.

However, the significant differences between the full inclusion and the adapted enrichment groups was also grounded in the perceptual IQ which is considered of fluid type, mainly, in Block design and Matrix. These two tests are used as measures of IQ in the abbreviated version of the WAIS^™^ [52] in population with typical development [53] as well as in a population with ID [54]. The students in the full inclusion model exhibited higher scores in the Hebrew fluid tests [16], including idioms, analogies and phonemic fluency which require a higher level of abstract thinking beyond the verbal ability acquired through the environment and are considered as fluid type.

The gap in fluid tests (perceptual IQ and the fluid Hebrew tests), indicate that the variance between the students with ID in the included and the adapted models was more than culture-dependent (residence and learning environment), rather it reflects the basic dilemma of intelligence theory [25, 26] and the nature of ID. Although crystallized intelligence is acquired and impacted by the environment, scientist claim that fluid intelligence influence the crystallized intelligence as well [49, 55]. That is, the degree of environment influence on intelligence depends on fluid intelligence. Thus, the difference between the students with ID in full inclusion and adapted enrichment models are more "structural" than developmental, and stem from individual differences in the *g–*the basic general intelligence [49] between the two groups [48, 56]. However, the higher fluid intelligence, mainly general intelligence, of students with ID in the full inclusion model, served as their **additional cognitive resource** which enables them to be fully included in undergraduate courses, as compared to the students with ID in the adapted group.

### Screening tool for determining eligibility for full inclusion of students with ID

The differences in the various cognitive tests between the students with ID in the full inclusion versus the adapted enrichment groups raised a clinical question: Which of the intelligence tests and the crystallized and/or fluid battery could serve as a screening tool for determining the appropriate placement of students with ID into the full inclusion group as opposed to the adapted enrichment models?

For this purpose, we used the ROC analysis, which differentiates between the groups in term of AUC sensitivity and specificity [45]. Of 25 intelligence measures (including the indexes and cognitive battery), seven could serve as screening tools for determining the appropriate placement of students with ID to the full inclusion group and the adapted enrichment models in terms of their AUC, sensitivity and specificity, which could not be captured by the MANOVA’s. These variables were: General IQ, verbal IQ, vocabulary, knowledge, and the comprehension index as well as synonyms (crystallized test) and idioms (fluid test).

Table 4 indicates that of the 10 students with ID in the full inclusion model, nine scored on or above the ROC cutoff point in all tests. One student—a woman with Down syndrome, scored below the cutoff points (IQG = 59, IQV = 61, vocabulary = 2, comprehension index = 67, knowledge = 5, synonyms = 4, idioms = 13). Although she did not fail in any of the courses, she revealed a lower level of understanding in the undergraduate courses and needed more mediation in terms of time and learning strategies. Furthermore, her scores in two courses (Introduction to Special Education and Intellectual Disability) were low compared to the other students with ID. Another student, also a woman with Down syndrome, received one point under the cutoff in synonyms, but in all the other tests her scores were at or above the cutoff point (IQG = 68, IQV = 66, vocabulary = 5, comprehension index = 67, knowledge = 7, synonyms = 4, idioms = 9). She also followed the courses’ requirements, did not fail any course and exhibited creativity in different domains.

It is noteworthy that the lower scores of two students with Down syndrome on some of the verbal tests is correlated with the cognitive profile of this etiology. Wang and Bellugi [50] found that individuals with Williams syndrome exhibit strength in verbal tasks and deficit in visual-spatial tasks. Contrastingly, individuals with Down syndrome exhibited deficit in verbal tasks, but preserved visual-spatial tasks [57]. However, the level of understanding of individuals with Down syndrome is higher than their production ability [58]. This feature of participants with Down syndrome might serve as an explanation for the fact that despite their lower verbal scores in accordance with the cutoff score, these two students did not fail any of the course requirements. This may be due to additional mediation that was provided to them during the year by the special education teacher as well as their higher motivation to succeed. We currently face the ethical dilemma of whether to keep the woman who scored below the cutoff point in all seven tests in the full inclusion program, which is time and money consuming. The other option is to allow her to study in undergraduate courses without the requirements. From an educational point of view, the results of the ROC analysis can serve as an operative tool for determining the amount and content of mediation that should be provided to the students with ID in the full inclusion model.

The general IQ and idioms scores seem to be the best indicators for determining whether a student was suitable for the adapted enrichment or the full inclusion program. Based on their cutoff scores, either none (for general IQ) or one (for idioms) student can move to the full inclusion model. On the other tests, the scores of 3–4 students were above the cutoff point, but those were not the same individuals, and each exhibited strengths and weaknesses in different tests.

In conclusion, this study was a first attempt to predict the appropriate placement of individuals with ID for undergraduate courses. This issue should be researched cautiously, thoroughly and longitudinally. The ROC analysis assists in the evaluation of the eligibility of students with ID for adapted enrichment versus full inclusion. One important conclusion that emerged from this study is associated with the current DSM-5 [11] definition of ID. As stated, IDD (Intellectual Developmental Disorder) is a disorder with onset during the developmental period that includes both intellectual and adaptive functioning deficits in conceptual, social, and practical domains. According to DSM-5, the deficits in intellectual functions of persons with ID lie in reasoning, problem-solving, planning, abstract thinking, judgment, **academic learning** and learning from experience. In light of the Empowerment Project and the results of the current study, it can be said that under certain mediation and support circumstances, students with ID are able to actively participate in undergraduate academic learning and achieve academic goals. However, amendment of this DSM-5 claim can be possible only after our students complete their courses for the BA degree.

This study raises several educational dilemmas. The first is related to the type of undergraduate courses in which adults with ID could be included. Currently, these individuals have succeeded in the first two years of social science courses in an undergraduate education program, which are based on crystallized abilities. Will they be able to cope with third year courses? This question remains unanswered at present.

### Attitudes towards the program of participants in the included model

The semi-structured interviews revealed several themes that express the attitudes of the students with ID in the inclusion model towards the program. We did not use any formal measure of quality of life but the interviews allowed us a glimpse at the students with ID’s thoughts and feelings towards their academic life. We analyzed the results according to Shkedi’s [43] three phases of qualitative research. In the "mapping stage" (see Results section) we categorize the answers of the participants into cognitive, emotional and motivational components of attitudes [46, 47]. The themes that emerged from the theoretical stage grounded the answers of the participants in theories of motivation [59, 60, 61] and self-determination [62].

Motivation is defined as a process that initiates, guides and maintains goal oriented behaviors or activates, directs, and sustains, even though the task involves barriers and obstacles [62]. Motivation emphasizes the universal will towards growth and development [62] and is associated with energy, direction, persistence, intention and activation and involves biological, social, and cognitive forces of the behavior. Maeher and Midgley [61] related to three components of motivation in learning: *Activation* which involves the decision to initiate (or to maintain) a behavior and continued effort towards a goal, despite the potential existence of obstacles. *Persistence* is the amount of time, energy and resources that are invested (behavior). *Intensity* (also known as quality) relates to the thought (cognition) and emotion put towards the goal (there are other interpretations to this concept).

Self-determination theory *(SDT)* [62] is a macro theory of human motivation and personality that deals with an individual’s inherent growth tendencies and innate psychological needs. It is concerned with the motivation behind choices made without external influence and interference.

It seems that the students with ID included in undergraduate courses were characterized by higher motivation and self-determination. They were self-determined to continue in the program despite the difficulties and to pay the ensuing "cost". They invested time and energy in the program, not only at the university, but at home, because they were aware of the cognitive and emotional contribution of participating in the program (Table 6).

The need for appreciation and respect in Maslow [59, 60] is based on the hierarchal motivational theory. When the needs at the bottom three levels have been satisfied (physical needs, safety, esteem and belonging) are fulfilled, it becomes increasingly important to gain the respect and appreciation of others. The answers of the students with ID (Table 6) indicate that learning in undergraduate courses and achieving academic accomplishments, played a role in fulfilling their esteem needs, the confidence in their abilities, and empowerment of their self-image.

The last need in the hierarchy is self-actualization, or the desire to fulfill one’s individual potential. "What a man can be, he must be," Maslow explained ([60], pp.203), referring to the need of people to achieve their full potential as human beings. Our study indicated that despite the limitations imposed by their disability, the concept "self-actualization" can be expanded to include people with ID. Learning at the university in undergraduate courses brought the students to a level of functioning previously absent from their behavioral repertoire, from both a cognitive and emotional point of view and helped them to facilitate their human potential and fulfill their self-actualization.

#### Limitations and future research

Generalization from this research should be regarded with caution, due to the small sample size and the specific undergraduate courses that were studied in our program. However, the Empowerment Program is the first of its kind (students with ID are expected to fulfill the requirements of undergraduate courses and receive academic credits) in Israel and the world over. This was, therefore, the largest sample that could be found. Further research using other screening tests, such as working memory and long-term memory, are needed in order to examine the criteria for participation of students with ID in the full inclusion model. In this study, we use a semi-structured interview in order to learn about the attitudes of students with ID towards the inclusion program. It is recommended to use other formal measures to examine other personal characteristics such as: motivation, curiosity, creativity and emotional intelligence as well as quality of life. The appropriateness of the criteria should be tested in longitudinal studies, where participants will be followed along their academic studies to discover the number of courses they can take simultaneously, the type of courses and the support system and mediation necessary to help them succeed. Our study focuses on social sciences. It is recommended to examine whether the tests that were used in the current study are suitable for other academic programs for adults with ID: such as technical college or liberal arts college.

#### Educational implications

All the students with ID in the full inclusion program live at home. The division of ID in the Welfare Ministry should take this fact into account and consider the provision of a more home-oriented environment to people with ID living in community residences, in order to facilitate participation in postsecondary education in academic settings.

Three students in the full inclusion model studied in special education schools and the other four in mainstream schools. All the students in the adapted enrichment group studied in special education schools. The Israeli Special Education Act 5748–1988 [63]; 5755–2005 [64], following the United States “Education for All Handicapped Children Act” [65], recommends inclusion of students with special needs in traditional classrooms, in a less restrictive environment. ID is the second most common disability after learning disability (19,559 students with learning disability and 12,382 students with ID) [66] but only 5% of students with mild ID are included in regular schools [66]. The educational curriculum and transition curricula are directed towards preparation of adolescents (16–21) with ID for life as adults focusing on adaptive behavioral, instrumental and vocational skills. PSE all over the world and in Israel indicate that adults with mild ID can benefit from academic learning and a portion can even be fully included into undergraduate courses. The education system should therefore construct an appropriate syllabus for this population, both in mainstream schools and within special education classes, which will be more cognitively-oriented in order to allow more students to study further in a college or university for at least one year (stages 1–2) and to fulfill their potential by being included in undergraduate courses.

## Supporting information

S1 Appendix Preparatory program according to the Universal learning design. (DOCX)
