# Supplementary material for: Crystallized and fluid intelligence of university students with intellectual disability who are fully integrated versus those who studied in adapted enrichment courses
Source: PLoS One. 2018 Apr 23;13(4):e0193351. doi: 10.1371/journal.pone.0193351 (PMC5912745; doi:10.1371/journal.pone.0193351)
Supplement: S1 Appendix — (DOCX) [file pone.0193351.s001.docx]

| **Appendix 1**  **Means of Representation** | | |
| --- | --- | --- |
| *Providing options for comprehension and vocabulary* | | |
| **Task** | **Cognitive Processes** | **Strategies** |
| Comprehension of the auditory content of the lectures | Supply background knowledge, cognitive expansion and highlighting pattern, critical features, ideas, theories. synthesizing, analyzing and drawing conclusions | Provide options for executive function: guide appropriate goal setting, support planning and strategies development, enhance capacity for monitoring of development progress, develop working and long term strategies in all the academic tasks |
| Reading comprehension of text | Clarify syntax and structure  Support decoding of text, titles, new words,  keywords words, | Small pedagogical units, simplification of information, appropriate language, concretization (using videos, pictures, reading materials, etc.), and making associations with previous knowledge |
| *Providing option for perception* | | |
| Power-point comprehension | Guide information processing, visualization and manipulation, Accessibility of the text according of Easy to Read strategies (Department of Health, 2010**)** | Use of videos, clips, pictures, reading materials |
| **Means Action and Expression** | | |
| *Providing options for expressions* | | |
| Studying and performing examinations | Coping with various types of exams: open- ended questions, multiple choice questions, planning, organizing and implementing of previous knowledge, abstraction and expansion, comparing, drawing conclusions |  |
| Writing assignments | Academic writing, basing arguments on literature, critical thinking |  |
| Research tasks (meta-cognition) | Meta-cognitive processes  Comprehend and analyze scientific articles and conducting research (research question, plan, measurement of tools, using questionnaires, analysis of results drawing conclusions and presenting findings) | Posit a research question, planning, understanding measurement tools, using questionnaires, analyzing findings, drawing conclusions and presenting findings |
| *Providing options for activity* | | |
| Self-management and self- searching skills | Search for skills in the text, search the web, do class work, homework, focus on main topics, read and summarize written material, search for the correct answer to questions | Varying methods of response and navigation,  Optimizing access to tools, technology.  Computers, IPAD, Tablet |
| **Means of Engagement** | | |
| Social interaction | Group work on tasks, discussions, facilitate personal ability to cope with the other students and foster collaboration and community, provide personal examples | Group work on tasks, discussing, asking and answering questions,  Planning, organizing, cognitive abstraction, and drawing conclusions |
| Providing options for self- regulation | Promote expectations and belief that optimize motivation, facilitate personal coping skills and strategies, develop self-assessment and reflection, |  |
| Providing options for recruiting interest | Optimizing individuals choice and autonomy, optimizing relevance and autonomy |  |
